# Supplementary material for: Federated analysis of autosomal recessive coding variants in 29,745 developmental disorder patients from diverse populations
Source: Nat Genet. 2024 Sep 23;56(10):2046–53. doi: 10.1038/s41588-024-01910-8 (PMC11525179; doi:10.1038/s41588-024-01910-8)
Supplement: Supplementary file 1 — Supplementary Methods, Supplementary Note, Supplementary Figs. 1–12. [file 41588_2024_1910_MOESM1_ESM.pdf]

nature genetics

Supplementary information

https://doi.org/10.1038/s41588-024-01910-8

Federated analysis of autosomal recessive coding variants in 29,745 developmental disorder patients from diverse populations

In the format provided by the authors and unedited

Supplementary Note **2**
Supplementary Methods **2**
Relatedness estimation 2
ROH calling 2
Variant QC 2
SNV QC 2
Indel QC 3
Sample QC 4
Filtering of missense and other functional variants 5
Filtering and analysis of de novo mutations 5
Burden explained by ClinVar pathogenic variants 6
Phenotypic similarity of patients 6
Supplementary Results **7**
Phenotypic comparisons of the cohorts 7
Contribution of multi-gene causes in DDD and GeneDx 7
Additional information on the genes in Table 2 8
CRELD1 *8*
KBTBD2 *9*
ZDHHC16 *10*
HECTD4 *10*
ATAD2B *11*
References **13**
Supplementary Figures **17**
Supplementary Figure 1 17
Supplementary Figure 2 18
Supplementary Figure 3 19
Supplementary Figure 4 20
Supplementary Figure 5 21
Supplementary Figure 6 21
Supplementary Figure 7 24
Supplementary Figure 8 25
Supplementary Figure 9 26
Supplementary Figure 10 27
Supplementary Figure 11 28
Supplementary Figure 12 29

Supplementary Note

Supplementary Methods

Relatedness estimation

Relatedness between samples within each cohort was estimated using KING (v2.2) –kinship[1]. For DDD, we used common variants (MAF>0.01) that passed the following hard genotype filters: genotype quality (GQ) > 20, depth (DP) > 7, p-value from a binomial test on allelic depth > 0.001, and (after applying those genotype-specific filters) low missingness (<5%). For GeneDx, we used the same set of SNPs as used in the PCA, described in the main Methods. We used a cutoff of kinship coefficient > 0.04419417 to define related individuals, which is the lower bound cutoff for third-degree relatives. For each cohort, a list of unrelated parents and unrelated probands was created in a way that maximized the number of samples retained.

ROH calling

Runs of homozygosity (ROHs) were called using bcftools-roh[2] using common variants (MAF>0.01) with GQ⩾20 and DP⩾7 and low genotype missingness (<10%). Our previous work noted the effect of LD thinning on the calling of ROHs, with the optimal LD thinning differing by autozygosity levels[3]. We repeated the ROH calling for four values of LD thinning, r^2={0.2,0.4,0.6,0.8}. The ROHs were called within each cohort for each GIA group independently to give a more accurate allele frequency estimate for the common variants used in the analysis. We retained ROHs that had quality score PHRED⩾20. For each individual, we calculated the fraction of the genome in runs of homozygosity, F_{ROH}. The distribution of F_{ROH} values for each GIA sub-group is shown in **Supplementary Figure 7**.

Variant QC

Autosomal SNVs and indels underwent quality control (QC) separately within each cohort. In brief, we restricted to the variants within the intersection of the calling regions of the two cohorts, calculated metrics to determine the quality of SNV and indels passing a set of different thresholds, then selected the thresholds so as to optimise these quality metrics (**Supplementary Figures 3 and 4**). This is now described in more detail below. The QC was conducted using bcftools version 1.16[4].

SNV QC

The following genotype- and variant-level metrics were tested:

Genotype-level metrics:
1. Genotype Quality (GQ > {20,25,30})
2. Depth (DP > {7, 10})
3. Binomial p-value of allelic depth (for heterozygotes) (P(AD) > {0, 0.001})

Variant-level metrics:
1. VQSLOD (VQSLOD > {-1.5, -2.0, -2.5, -3.0})

2. Fraction of non-missing genotypes passing genotype-level QC thresholds (FPASS > {0.5, 0.7})

For each combination of metrics, we measured the following:

1. Transmission rate of synonymous singletons. Specifically, we identified synonymous variants seen in a single parent in the dataset, and determined what fraction of these were transmitted to the child. Since these variants are unlikely to contribute to the phenotype, we expect them to be transmitted 50% of the time, so we optimised the choice of QC parameters to get this metric as close as possible to 50% while simultaneously optimising the other metrics (**Supplementary Figure 3**).
2. Sensitivity to detect known *de novo* SNVs. Previous analysis had identified a total of 41,890 (38,038 SNV, and 3,852 indel) likely pathogenic *de novo* mutations across the two cohorts that passed stringent quality control[5]. We wanted to retain as many of these variants as possible (**Supplementary Figure 3**).
3. Number of variants that are called as homozygous for the alternate allele in the child and homozygous for the reference allele in both parents. These candidate *de novo* mutations are almost certainly errors.
4. Number of candidate *de novo* mutations that are seen in multiple individuals across the dataset (recurrent *de novo* mutations), likely to be enriched for errors.
5. Rate of Mendelian errors in trios.
6. Total number of variants passing QC. We wish to maximise this while simultaneously optimising the other metrics.
7. The transition to transversion ratio Ts/Tv (**Supplementary Figure 3**).

We chose the following thresholds for SNV QC:
For DDD:

- GQ > 20
- DP > 7
- P(AD) > 0.001
- VQSLOD > -2.0
- FPASS > 0.5

For GeneDx:

- GQ > 25
- DP > 10
- P(AD) > 0.001
- VQSLOD > -2.0
- FPASS > 0.7

Indel QC

The following genotype- and variant-level metrics were tested:

Genotypes-level metrics:

1. Genotype Quality (GQ > {20,25,30})
2. Depth (DP > {7, 10})

3. Allelic Balance (Variant Allele Frequency) for heterozygotes (AB > {0.2, 0.3})

Variant-level metrics:

1. VQSLOD (VQSLOD > {-2.0, -5.0}, and no VQSLOD)
2. Fraction of genotypes passing genotype-level QC thresholds (FPASS > {0.5, 0.7}).

For each combination of metric we measured:

1. Transmission rate of rare inframe variants in low pLI, non-monoallelic DDG2P genes (**Supplementary Figure 4**). The logic here is the same as for the synonymous singletons mentioned above; inframe indels in these genes are likely under minimal selective pressure, on average, so we calibrated our QC so that the transmission of these variants was as close as possible to 50% while also optimising the other metrics. We also filtered to variants with MAF<0.001 in (or absent from) gnomAD[6] when checking this metric.
2. Sensitivity to detect *de novo* indels. A total of 3,852 *de novo* indel mutations were detected across the two cohorts in previous analyses[5]. We wanted to maximise our sensitivity to detect these mutations (**Supplementary Figure 4**).
3. Number of coding indels passing QC (**Supplementary Figure 4**).
4. Ratio of frameshift to nonsense variants. From previous studies[7,8], we expect the ratio of frameshift to nonsense mutations to be roughly 1.2.

We chose the following thresholds for indel QC:

For DDD:

- GQ > 20
- DP > 7
- AB > 0.2
- No VQSLOD
- FPASS > 0.5

For GeneDx:

- GQ > 30
- DP > 7
- AB > 0.3
- No VQSLOD
- FPASS > 0.7

The number of variants before and after QC is given in **Supplementary Table 3** and the distribution of the number of variants per proband is given in **Supplementary Figure 5**.

Sample QC

After applying the variant and genotype QC, we carried out sample QC by running regressions of different quality metrics on several covariates (detailed below), then removing individuals whose residuals were greater than four median absolute deviations from the median for these two regressions (**Supplementary Figure 6**). We ran these two regressions:

ratio of transitions to transversions ~ exome_capture_platform + GIA_subgroup_label

ratio_of_heterozygous_to_homozygous_genotypes ~ exome_capture_platform + GIA_subgroup_label + F_{ROH}

In addition, we removed individuals with a proportion of genotypes missing greater than 0.2.

The number of individuals in each GIA sub-group before and after QC is given in **Supplementary Table 2**.

Filtering of missense and other functional variants

There are many metrics to predict deleteriousness of missense variants, but most of these are focused on predicting deleteriousness in the heterozygous state. We assessed several of these metrics (VARITY[9], PrimateAI[10], MPC[11], CADD[12,13], ClinPred[14], MoI-Pred recessive probability[15], REVEL[16], PolyPhen[17]), comparing the distributions for known pathogenic recessive missense variants in DDD (i.e., annotated as pathogenic/likely pathogenic in DECIPHER) to all missense variants on chromosome 20 (**Supplementary Figure 11**). We observed that PrimateAI and MPC were less discriminating than the other metrics in this context, and thus removed them from consideration. (Although CADD was also less discriminating, we retained it since it was available for all variants, unlike some of the other annotations.) We also removed VARITY-R since it uses the same model as VARITY-ER but just a different training set. For each of the remaining six metrics, we defined the threshold which gave us 90% sensitivity to detect the known pathogenic recessive missense variants (CADD_PHRED⩾24.18; REVEL⩾0.36; VARITYER_LOO⩾0.25; PolyPhen⩾0.59; ClinPred⩾0.53; MoI-Pred recessive probability⩾0.11). For inframe indels, we used a filter of CADD_PHRED⩾17.34 (i.e., the CADD value which captures 90% of known inframe pathogenic recessive variants from DECIPHER[18]). For the remaining consequences that are included in the “functional” category (start_lost, transcript_amplification, protein_altering_variant, splice_region_variant, LoFs predicted to be low confidence (LC) by LOFTEE, and synonymous variants with a minimum SpliceAI score of 0.8), we use a filter of CADD_PHRED⩾24.18.

We then evaluated the burden (observed/expected) and attributable fraction ([observed-expected]/sample size) (see section on Burden Analysis in the main Methods) obtained for LoF/functional and functional/functional biallelic genotypes when requiring missense variants to pass the above cutoffs for different numbers of annotations (**Supplementary Figure 8**). Since not all of these deleteriousness metrics were available for all missense variants, we additionally evaluated the burden and attributable fraction when requiring missense variants to pass ⩾70% of available annotations (**Supplementary Figure 8**). This final filter was the one chosen for the main analyses, on the basis of giving relatively high observed/expected (i.e., more significant enrichment) as well as relatively high attributable fraction (i.e., explaining more probands).

Filtering and analysis of *de novo* mutations

De novo mutations (DNMs) were called by GATK Haplotype Caller, and variant calls were restricted to -/+ 50bp of RefGene primary coding regions. DNM calls were required to have greater than 10 reads in the proband, more than 3 supporting reads, a genotype quality of

greater than 40 and a strand bias of less than 30, and allele fraction of >0.15 for SNVs and >0.25 for indels (except for calls on chrX in males, which were allowed to have an allele fraction of 1). Indels greater than 100bp and variants that were seen in more than 11 parents across the cohort were removed. Sites with an allele fraction of less than 0.3 were excluded if any one of the following conditions were met: BaseQRankSum (Z-score from Wilcoxon rank sum test of Alt Vs. Ref base qualities) <= 0.75, MQ (RMS Mapping Quality) <= 58 or QD (Variant Confidence/Quality by Depth) <= 8. Variants were annotated with bcftools-csq[19] on the canonical transcript (Gencode GRCh38, version 43).

We calculated the expected number of DNMs in subgroupings of probands using a gene-specific null mutation rate model for different functional classes of mutations based on estimated triplet-specific mutation rates, accounting for gene length and sequence context[20]. The exome-wide attributable fraction was calculated as follows, for a given group of probands:

$$De\ novo\ attributable\ fraction\ = \frac{(O_{LoF} - \lambda E_{LoF}) + (O_{functional} - \lambda E_{functional})}{\#\ probands}$$

where O_c is the exome-wide observed number of *de novo* variants in consequence class *c*, E_c is the expected number calculated using the model from [20], and λ is a correction factor calculated as $\frac{O_{synonymous}}{E_{synonymous}}$.

Burden explained by ClinVar pathogenic variants

Figure 2b shows the estimate of the autosomal recessive attributable fraction after removing variants annotated as pathogenic/likely pathogenic (P/LP) in ClinVar. For this, we removed biallelic genotypes if the variant (for homozygotes) or both variants (in a compound heterozygote pair) fulfilled the following criteria:

- had CLNSIG=Pathogenic OR CLNSIG=Likely_pathogenic, OR
- had CLNSIG=Conflicting_interpretations_of_pathogenicity AND (CLNSIGCONF~Pathogenic|Likely_pathogenic AND NOT CLNSIGCONF~Benign|Likely_benign) i.e., if there were conflicting assertions of pathogenicity, at least one of those assertions was P/LP, but none were “benign” or “likely benign”

Phenotypic similarity of patients

The phenotypic similarity of patients was calculated following Kaplanis et al.[5] with the *phenopy* package https://github.com/GeneDx/phenopy. The pairwise similarity of two terms in the Human Phenotype Ontology (HPO) were compared quantitatively using the Hybrid Relative Semantic Similarity (HRSS) metric, and similarity for two lists of terms was calculated via a Best Match Average[21]. The phenotype similarity between two probands is defined as the listwise HRSS of the phenotypes describing each proband. Only terms currently in the HPO at the time of analysis were included, and any updates to retired HPO terms were handled by searching for the alternate IDs of all current phenotypes and replacing them where appropriate. The set of HPO terms assigned to each proband were pruned by removing ancestor terms in any ancestor-descendant pairs. As before, the information content (IC) of each term used for HRSS

calculations was the mean of the IC based on the HPO-OMIM-ORPHANET phenotype-to-gene annotations and the phenotype-to-gene annotations of monogenic diagnosed cases from the relevant cohort/s (i.e., DDD alone if examining a pair of DDD patients, GeneDx alone if examining a pair of GeneDx patients, or DDD+GeneDx if examining a pair consisting of one patient from each cohort).

For the genes that passed FDR<5% in the genotype-based test, the phenotypic similarity of all pairs of probands with damaging biallelic variants in that gene were calculated. We compared the distribution of these scores to a null distribution of HRSS scores for 100,000 randomly-chosen pairs (**Extended Data Figure 7**). This null distribution was created such that the fraction of randomly-chosen pairs that involved a) two DDD patients, b) two GeneDx patients or c) one DDD and one GeneDx patient matched the fraction amongst the patients with damaging biallelic variants in the FDR<5% genes.

Supplementary Results

Phenotypic comparisons of the cohorts

DDD patients were slightly more male-biased than GeneDx patients (58.4% male versus 55.7% male, Fisher's exact test p-value = $8x10^{-8}$). They were also slightly younger at recruitment on average (7.3 years versus 9.4 years; t-test p-value $<1x10^{-163}$), and the age distribution was less variable (standard deviation 6.1 years for DDD versus 10.2 years for GeneDx) (**Supplementary Figure 1**). DDD patients had significantly fewer HPO terms on average than GeneDx patients (7.0 terms versus 19.8; t-test p-value$<1x10^{-200}$). This likely reflects differences in how these HPO terms were recorded. For DDD, clinical geneticists recorded phenotypes that they thought likely to be relevant to a monogenic diagnosis and that were particularly distinctive amongst the population of rare disease patients being seen in genetics clinics, whereas in GeneDx, the HPO terms were extracted from the medical notes (including medical history and primary indication) through a mixture of automated text mining and manual curation by nurses, contractors and genetic counsellors trained in the abstraction process. Accordingly, there were multiple organ systems in which GeneDx patients were substantially more likely than DDD patients to have an HPO term, even after controlling for age and sex, including the musculature, digestive, cardiovascular, immune, respiratory and blood systems (**Supplementary Figure 2**). However, examination of the most common HPO terms revealed that many of those assigned to GeneDx patients are nonspecific (e.g., feeding difficulties, bruising susceptibility, failure to thrive) or represent common diseases (e.g., asthma, eczema) (**Supplementary Table 1**). These differences are likely related to differences in coding practices between the clinicians recruiting to DDD versus GeneDx rather than true phenotypic differences between cohorts.

Contribution of multi-gene causes in DDD and GeneDx

We used this joint DDD-GeneDx dataset to explore the contribution of multi-gene causes to these two cohorts. In DDD, we previously reported that 121 (2.7%) of the 4484 probands who had received a diagnosis by means of clinical assertion had two or more different genetic diagnoses[22], which was very similar to the proportion in GeneDx (237/9949, 2.4%). The

proportion in DDD increased to 359 out of 5502 (6.5%) diagnosed probands after additionally considering automated ACMG variant classifications[22]. However, these numbers might under-estimate the true number of multi-gene diagnoses within the cohorts, since phenotypic heterogeneity conferred by variants in the same gene may make it difficult to assess putatively damaging variants clinically. Thus, to investigate this further, we carried out exome-wide burden analyses of autosomal recessive and *de novo* variants to see whether there was any excess of these in diagnosed patients with single diagnoses (i.e., excluding known or predicted composite diagnoses) once the known diagnostic variants were removed (**Extended Data Figure 6**). We found no significant burden of damaging biallelic variants in probands with a *de novo* diagnosis or in probands with a recessive diagnosis once the diagnostic variants had been removed (attributable fractions 0.1% [-0.5-0.8%] and 0% [-7.6-8.7] respectively). However, we found a significant burden of damaging *de novo* mutations in patients with a *de novo* diagnosis, even after removing the diagnostic variants (attributable fraction 12.5% [9.6-15.5%]; ~502 individuals), as well as in patients with a diagnosis involving inherited dominant, recessive or X-linked variants (attributable fraction 12.5% [8.3-16.8%]; ~241 individuals). Almost all of the residual *de novo* burden in diagnosed individuals was outside of known monoallelic or X-linked dominant DDG2P genes (11.6% [9.3-14.0%], **Extended Data Figure 6B**). In DDD, we split the diagnosed patients into those classified by clinicians as having a full *versus* partial diagnosis, and noted that the residual burden of *de novo* mutations (after removing the diagnostic ones) was significantly different from 0 in the 'fully diagnosed' set (attributable fraction 5.7% [0.8-10.8%]) but was higher in the partially diagnosed set (attributable fraction 18.0% [3.4-34.5%]).

Additional information on the genes in Table 2

CRELD1

CRELD1 ($p=9.08 \times 10^{-8}$) is an established monoallelic (dominant) DD gene on both the DDG2P and GeneDx lists, in which heterozygous missense variants are associated with susceptibility to atrioventricular septal defects (AVSD)[23,24]. *CRELD1* (cysteine-rich with epidermal growth factor (EGF)-like domains 1) is involved in calcineurin/NFATc1 signaling during endocardial and myocardial development[25] and modulates homeostasis in the immune system[26]. However, in humans, it is also highly expressed in developing brain, heart branchial arches, and limb buds[27]. We observed eight probands with biallelic LoF/functional or functional/functional genotypes in this gene, all of whom had global developmental delay and seizures, plus variable other features. A concurrent study involving patients from GeneDx and other cohorts has also identified *CRELD1* as a novel ARDD gene causing a multisystem syndrome including neurodevelopmental phenotypes in all patients, cardiac defects in 8/18 patients, plus variable other features[28]. Three of the five GeneDx patients we identified were also in that study.

Amongst 141,417 patients from GeneDx who were sequenced after the data freeze on which our main analysis was based, we subsequently identified four additional patients with biallelic variants in *CRELD1* that passed our filters. Of these, two had phenotypes that were similar to other biallelic *CRELD1* patients, and these are included in **Supplementary Table 6**. However, two had phenotypes that were distinct from other *CRELD1* patients (notably, no developmental

delay or seizures). For one of these, their phenotype seemed better explained by variants in another gene which had been previously reported and deemed diagnostic. The other had a partial diagnosis in another gene but since their phenotype does not fit well with the other *CRELD1* cases, we would class their *CRELD1* variants as VUSs. For both of these probands, their *CRELD1* genotypes involved missense variants that had not been previously seen in any of the other cases and these may well be benign, despite passing our filters (chr3:9982683:C:T; ENST00000326434.5:c.610C>T; ENSP00000321856.5:p.Arg204Cys and chr3:9984790:G:A; c.847G>A; p.Gly283Arg).

Of the ten patients with biallelic *CRELD1* variants that we think are likely to be causal, all have genotypes involving missense variants that disrupt cysteine residues in or around the EGF-like and calcium-binding EGF-like domains, so they may destroy disulfide bonding, as noted in Jeffries *et al.*[28]; specifically, seven have genotypes involving the p.Cys192Tyr variant Jeffries *et al.* reported as recurrent (of which two were previously reported in their paper), one has p.Cys262Arg *in trans* with a pLoF (patient also reported by Jeffries *et al.*), and two siblings are homozygous for p.Cys218Tyr. The four additional missense variants reported in Jeffries *et al.* were all in the transmembrane domains at the C-terminal end of the protein. In contrast, the missense variants that have been reported to predispose to AVSD do not show any particular spatial clustering, and none involve cysteine residues. Further functional work would be required to definitively establish the molecular consequences of missense variants contributing to the recessive *CRELD1* neurodevelopmental disorder versus those predisposing to AVSD in the heterozygous state.

KBTBD2

Having identified two patients with rare LoF/missense compound heterozygous variants in our original GeneDx sample (described in the main text), yielding a Bonferroni-significant p-value, we subsequently identified a female patient in the CENTOGENE Biodatabank who had a homozygous frameshift variant in this gene (c.1298_1299delTG; p.Val433Aspfs*14) (**Supplementary Table 6**). This variant is absent in gnomAD and was only detected in this individual amongst nearly 900,000 individuals in the CENTOGENE Biodatabank. The patient was born at 39 weeks to consanguineous parents, and had intrauterine growth restriction (IUGR), with a birth weight of 1 kg. Dysmorphic features were noted (low set ears, microphthalmia, micrognathia). She presented with microcephaly, cataracts, fisted clenched hands, and rocker bottom feet. She died at the age of three months. No hypoglycemia or features suggestive of diabetes could be documented. It is notable that this individual, with a homozygous LoF, had a more severe phenotype (i.e., very early death) than our two original patients, who had compound heterozygous LoF/missense variants, one of whom died in later infancy and the other was recruited as an adolescent. Two female siblings of the index Centogene patient died in infancy and had a very similar phenotype. Specifically, the first affected sister presented with IUGR, dysmorphism, bilateral corneal haziness and contracture of the joints of the upper and lower limbs. Upon ophthalmic exam, congenital glaucoma was confirmed. She presented with congenital heart disease (pulmonary stenosis and hypertrophic cardiomyopathy) and died at the age of two months. The second sister presented with IUGR,

dysmorphic features, rocker bottom feet, clenched hands, microphthalmia, micrognathia, low-set ears, bilateral corneal haziness, aniridia, pulmonary stenosis, hypertrophic cardiomyopathy and transaminitis. She died at the age of one month. The genotypes of these two siblings are not known, but it seems highly plausible that they may also have been homozygous for this private frameshift variant.

ZDHHC16

Our signal in *ZDHHC16* was driven by one patient with a LoF/LoF genotype and two with LoF/functional genotypes ($p=3.04x10^{-6}$ when including both diagnosed and undiagnosed probands, $p=6.05x10^{-7}$ when using undiagnosed probands only). Two of these unrelated probands were strikingly similar phenotypically, having microcephaly, seizures, developmental regression/neurodegeneration, and abnormalities of the respiratory system. However, the third had a less distinctive phenotype involving generalised developmental delay with seizures, and had a sibling with a similar phenotype and the same *ZDHHC16* variants. Amongst 141,417 patients from GeneDx who were sequenced after the data freeze on which our main analysis was based, we subsequently identified two more patients with biallelic damaging variants in this gene that would pass our filtering, one with compound heterozygous LoFs and one with a homozygous missense variant (**Supplementary Table 6**). Both of these patients had seizures, abnormalities of the respiratory system, and structural brain abnormalities. One had microcephaly, and one had neurodegeneration.

We subsequently also identified a male patient from Centogene with a homozygous variant affecting the canonical splice donor site (c.1019+2T>C) which is predicted to lead to exon skipping (**Supplementary Table 6**). This variant is absent in gnomAD and was only detected in this individual in the CENTOGENE Biodatabank. The patient was born prematurely to consanguineous parents. He presented with epileptic encephalopathy at the age of two months. He had microcephaly, severe axial hypotonia with hyperreflexia and spasticity, talipes equinovarus, dysmorphic features (curved eyebrows, full cheeks, nasal hypoplasia, micrognathia) and inguinal and umbilical hernias. He failed to thrive, currently receives feeding via a nasogastric tube, is unable to walk and has not developed any language. Brain MRI showed cerebellar hypoplasia. Although he did display laryngomalacia and developmental regression, similar to two of the original patients, the significance of these is difficult to assess in the context of his hypotonia and epileptic encephalopathy.

Thus, in summary, of these seven patients identified with damaging biallelic variants in *ZDHHC16*, all had seizures, four had microcephaly, four had developmental regression/neurodegeneration, and five had abnormalities of the respiratory system. Consistent with the neurodevelopmental features in these patients, *ZDHHC16* has been shown to play a critical role in the regulation of neural stem/progenitor cell proliferation in zebrafish telencephalic development[29]. It is part of a co-expression module enriched in human fetal brain[30], but further work is needed to elucidate its function during human brain development.

HECTD4

Biallelic variants in *HECTD4* (p=8.80x10^{-6} in our analysis) were recently reported to cause a neurodevelopmental disorder characterised by intellectual disability, seizures, movement disorder, behavioural abnormalities, macrocephaly, abnormality of dentition, and agenesis of the corpus callosum[31]. The two unrelated probands we observed with biallelic LoF/LoF genotypes in this gene also exhibited many of these features. We also subsequently identified two more GeneDx patients with damaging biallelic variants in this gene, including one with a homozygous stop gain variant and one with compound heterozygous predicted deleterious missense variants. Both had abnormalities of the corpus callosum and one had seizures.

HECTD4 encodes a ubiquitin E3 ligase in the HECT E3 family, is part of a co-expression module enriched in human fetal brain[30], and shows particularly high expression in the cerebellum in adults (https://www.gtexportal.org/home/gene/HECTD4). Variants in other members of the HECT E3 family are also known to cause neurodevelopmental disorders, including *UBE3A*, *HERC1*, *HERC2*, *HUWE1*, and *TRIP1*[32].

ATAD2B

Our signal in this gene was driven by two patients (p=1.02x10^{-5}), one with compound heterozygous LoFs and the other LoF/missense, of whom one had an affected sibling with the same genotype and a similar phenotype. Amongst the new set of GeneDx patients mentioned above, we subsequently identified a fourth patient who had biallelic variants passing our filtering, specifically compound heterozygous predicted damaging missense variants. All four of these patients had developmental delay or cognitive impairment (which are present in most patients in our study) and there was no particularly striking phenotypic similarity between them, other than between the two siblings (**Supplementary Table 6**).

One of the four patients was classified as “diagnosed” using automated ACMG criteria as part of the DDD study [22], so this patient was dropped when we restricted the gene-discovery analysis to undiagnosed patients, explaining the much higher p-value we then obtained (p=2.81x10^{-3}). However, this diagnosis has not been confirmed clinically. The putatively pathogenic variant was a *de novo* splice region variant in a “DDG2P limited” gene, which passed the DDD clinical filtering pipeline at the time but would fail in the current pipeline (which ignores splice region variants given the limited evidence that they cause loss-of-function, and genes with only limited evidence for disease association). It passed the automated ACMG filtering primarily because it is a *de novo* with a low MAF, pushing the posterior probability > 0.9. The gene is linked to a muscle disorder, which may explain part of this patient’s phenotype, but would not explain the neurodevelopmental features. Hence, we consider this diagnosis very tentative, and partial at best. It is possible that the biallelic damaging variants in *ATAD2B* are part of a dual diagnosis in this patient, although more evidence is needed to confirm this.

ATAD2B encodes a conserved nuclear protein that has been shown to be highly expressed during neuronal differentiation in chicken [33] and in human fetal brain (https://www.ebi.ac.uk/gxa/genes/ENSG00000119778?bs=%7B%22homo%20sapiens%22%3A%5B%22ORGANISM_PART%22%5D%7D&ds=%7B%22kingdom%22%3A%5B%22animals%2

2%5D%7D). Its bromodomain is involved in recognition of mono- and diacetylated histones[34], which is notable since many other genes involved in histone modification are known neurodevelopmental disorder genes[35]. A homozygous mouse knockout of *Atad2b* shows behavioural abnormalities[36]. Furthermore, this gene has been implicated as the most likely causal gene at a locus identified through genome-wide association studies for intelligence[37] and educational attainment[38]. Finally, *ATAD2B* is highly constrained, with a pLI score of 1[7], indicating that heterozygous LoFs are under strong negative selection. Since it does not appear to cause a severe dominant disorder (parents with heterozygous LoFs were clinically unaffected), it seems likely that biallelic loss-of-function may confer a severe phenotype.

All in all, there is considerable circumstantial evidence that biallelic disruption of *ATAD2B* may perturb neurodevelopment. However, given the only moderate statistical evidence and lack of striking phenotypic similarity between the patients, we believe more evidence is needed to implicate this as a definitive ARDD gene.

References

1. Manichaikul, A. *et al.* Robust relationship inference in genome-wide association studies. *Bioinformatics* **26**, 2867–2873 (2010).
2. Narasimhan, V. *et al.* BCFtools/RoH: a hidden Markov model approach for detecting autozygosity from next-generation sequencing data. *Bioinformatics* **32**, 1749–1751 (2016).
3. Martin, H. C. *et al.* Quantifying the contribution of recessive coding variation to developmental disorders. *Science* **362**, 1161–1164 (2018).
4. Danecek, P. *et al.* Twelve years of SAMtools and BCFtools. *Gigascience* **10**, (2021).
5. Kaplanis, J. *et al.* Evidence for 28 genetic disorders discovered by combining healthcare and research data. *Nature* **586**, 757–762 (2020).
6. Karczewski, K. J. *et al.* The mutational constraint spectrum quantified from variation in 141,456 humans. *Nature* **581**, 434–443 (2020).
7. Lek, M. *et al.* Analysis of protein-coding genetic variation in 60,706 humans. *Nature* **536**, 285–291 (2016).
8. Van Hout, C. V. *et al.* Exome sequencing and characterization of 49,960 individuals in the UK Biobank. *Nature* **586**, 749–756 (2020).
9. Wu, Y., Li, R., Sun, S., Weile, J. & Roth, F. P. Improved pathogenicity prediction for rare human missense variants. *Am. J. Hum. Genet.* **108**, 1891–1906 (2021).
10. Sundaram, L. *et al.* Predicting the clinical impact of human mutation with deep neural networks. *Nat. Genet.* **50**, 1161–1170 (2018).
11. Samocha, K. E. *et al.* Regional missense constraint improves variant deleteriousness prediction. *bioRxiv* (2017) doi:10.1101/148353.
12. Rentzsch, P., Schubach, M., Shendure, J. & Kircher, M. CADD-Splice-improving genome-wide variant effect prediction using deep learning-derived splice scores. *Genome Med.* **13**, 31 (2021).

13. Rentzsch, P., Witten, D., Cooper, G. M., Shendure, J. & Kircher, M. CADD: predicting the deleteriousness of variants throughout the human genome. *Nucleic Acids Res.* **47**, D886–D894 (2019).

14. Alirezaie, N., Kernohan, K. D., Hartley, T., Majewski, J. & Hocking, T. D. ClinPred: Prediction Tool to Identify Disease-Relevant Nonsynonymous Single-Nucleotide Variants. *Am. J. Hum. Genet.* **103**, 474–483 (2018).

15. Petrazzini, B. O. *et al.* Prediction of recessive inheritance for missense variants in human disease. *medRxiv* 2021.10.25.21265472 (2021) doi:10.1101/2021.10.25.21265472.

16. Ioannidis, N. M. *et al.* REVEL: An Ensemble Method for Predicting the Pathogenicity of Rare Missense Variants. *Am. J. Hum. Genet.* **99**, 877–885 (2016).

17. Adzhubei, I. A. *et al.* A method and server for predicting damaging missense mutations. *Nat. Methods* **7**, 248–249 (2010).

18. Firth, H. V. *et al.* DECIPHER: Database of Chromosomal Imbalance and Phenotype in Humans Using Ensembl Resources. *Am. J. Hum. Genet.* **84**, 524–533 (2009).

19. Danecek, P. & McCarthy, S. A. BCFtools/csq: haplotype-aware variant consequences. *Bioinformatics* **33**, 2037–2039 (2017).

20. Samocha, K. E. *et al.* A framework for the interpretation of de novo mutation in human disease. *Nat. Genet.* **46**, 944–950 (2014).

21. Wu, X., Pang, E., Lin, K. & Pei, Z.-M. Improving the measurement of semantic similarity between gene ontology terms and gene products: insights from an edge- and IC-based hybrid method. *PLoS One* **8**, e66745 (2013).

22. Wright, C. F. *et al.* Genomic Diagnosis of Rare Pediatric Disease in the United Kingdom and Ireland. *N. Engl. J. Med.* **388**, 1559–1571 (2023).

23. Robinson, S. W. *et al.* Missense mutations in CRELD1 are associated with cardiac atrioventricular septal defects. *Am. J. Hum. Genet.* **72**, 1047–1052 (2003).

24. Zatyka, M. *et al.* Analysis of CRELD1 as a candidate 3p25 atrioventicular septal defect

locus (AVSD2). *Clin. Genet.* **67**, 526–528 (2005).

25. Mass, E., Wachten, D., Aschenbrenner, A. C., Voelzmann, A. & Hoch, M. Murine Creld1 controls cardiac development through activation of calcineurin/NFATc1 signaling. *Dev. Cell* **28**, 711–726 (2014).
26. Bonaguro, L. *et al.* CRELD1 modulates homeostasis of the immune system in mice and humans. *Nat. Immunol.* **21**, 1517–1527 (2020).
27. Rupp, P. A. *et al.* Identification, genomic organization and mRNA expression of CRELD1, the founding member of a unique family of matricellular proteins. *Gene* **293**, 47–57 (2002).
28. Jeffries, L. *et al.* Biallelic CRELD1 variants cause a multisystem syndrome including neurodevelopmental phenotypes, cardiac dysrhythmias, and frequent infections. *Genet Med* **26**, 101023 (2024).
29. Shi, W. *et al.* ZDHHC16 modulates FGF/ERK dependent proliferation of neural stem/progenitor cells in the zebrafish telencephalon. *Dev. Neurobiol.* **76**, 1014–1028 (2016).
30. Li, M. *et al.* Integrative functional genomic analysis of human brain development and neuropsychiatric risks. *Science* **362**, (2018).
31. Faqeih, E. A. *et al.* Biallelic variants in HECT E3 paralogs, HECTD4 and UBE3C, encoding ubiquitin ligases cause neurodevelopmental disorders that overlap with Angelman syndrome. *Genet. Med.* **25**, 100323 (2023).
32. Wang, Y., Argiles-Castillo, D., Kane, E. I., Zhou, A. & Spratt, D. HECT E3 ubiquitin ligases – emerging insights into their biological roles and disease relevance. *J. Cell Sci.* **133**, (2020).
33. Leachman, N. T., Brellier, F., Ferralli, J., Chiquet-Ehrismann, R. & Tucker, R. P. ATAD2B is a phylogenetically conserved nuclear protein expressed during neuronal differentiation and tumorigenesis. *Dev. Growth Differ.* **52**, 747–755 (2010).
34. Lloyd, J. T. *et al.* Structural Insights into the Recognition of Mono- and Diacetylated Histones by the ATAD2B Bromodomain. *J. Med. Chem.* **63**, 12799–12813 (2020).
35. Fallah, M. S., Szarics, D., Robson, C. M. & Eubanks, J. H. Impaired Regulation of Histone

Methylation and Acetylation Underlies Specific Neurodevelopmental Disorders. *Front. Genet.* **11**, 613098 (2020).

36. Dickinson, M. E. *et al.* High-throughput discovery of novel developmental phenotypes. *Nature* **537**, 508–514 (2016).
37. Sniekers, S. *et al.* Genome-wide association meta-analysis of 78,308 individuals identifies new loci and genes influencing human intelligence. *Nat. Genet.* **49**, 1107–1112 (2017).
38. Lee, J. J. *et al.* Gene discovery and polygenic prediction from a 1.1-million-person GWAS of educational attainment. *Nat. Genet.* **50**, 1112 (2018).
39. Akawi, N. *et al.* Discovery of four recessive developmental disorders using probabilistic genotype and phenotype matching among 4,125 families. *Nat. Genet.* **47**, 1363–1369 (2015).

Supplementary Figures

Supplementary Figure 1

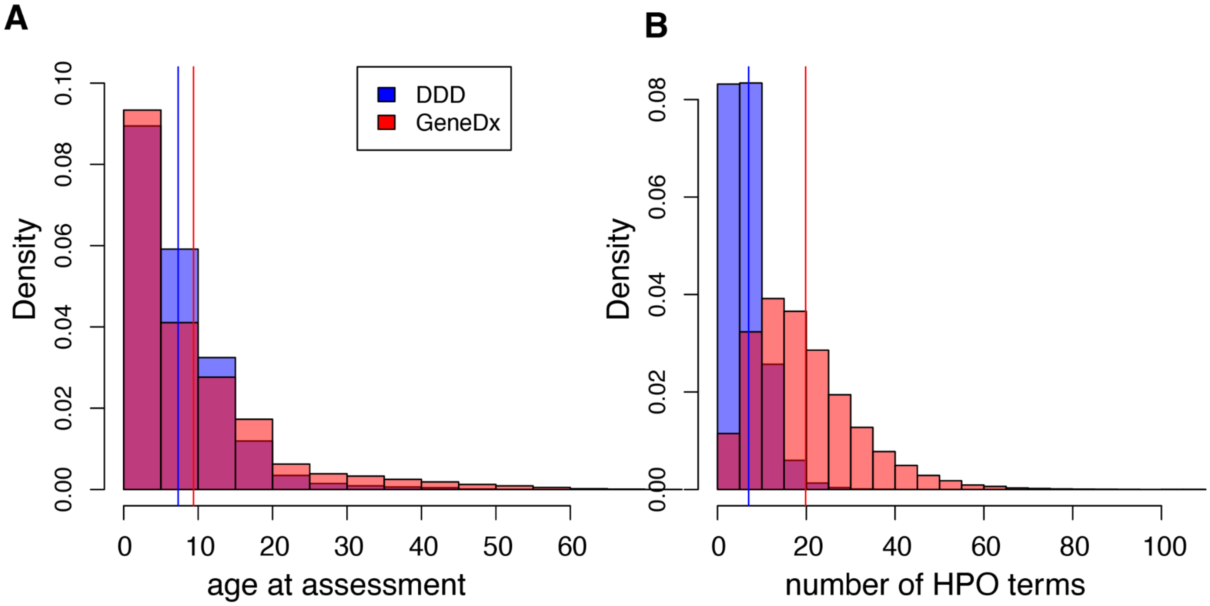

Supplementary Figure 1: Distribution of age at assessment (A) and number of HPO terms (B) between 13,450 DDD patients and 36,057 GeneDx patients. The vertical lines indicate the means.

Supplementary Figure 2

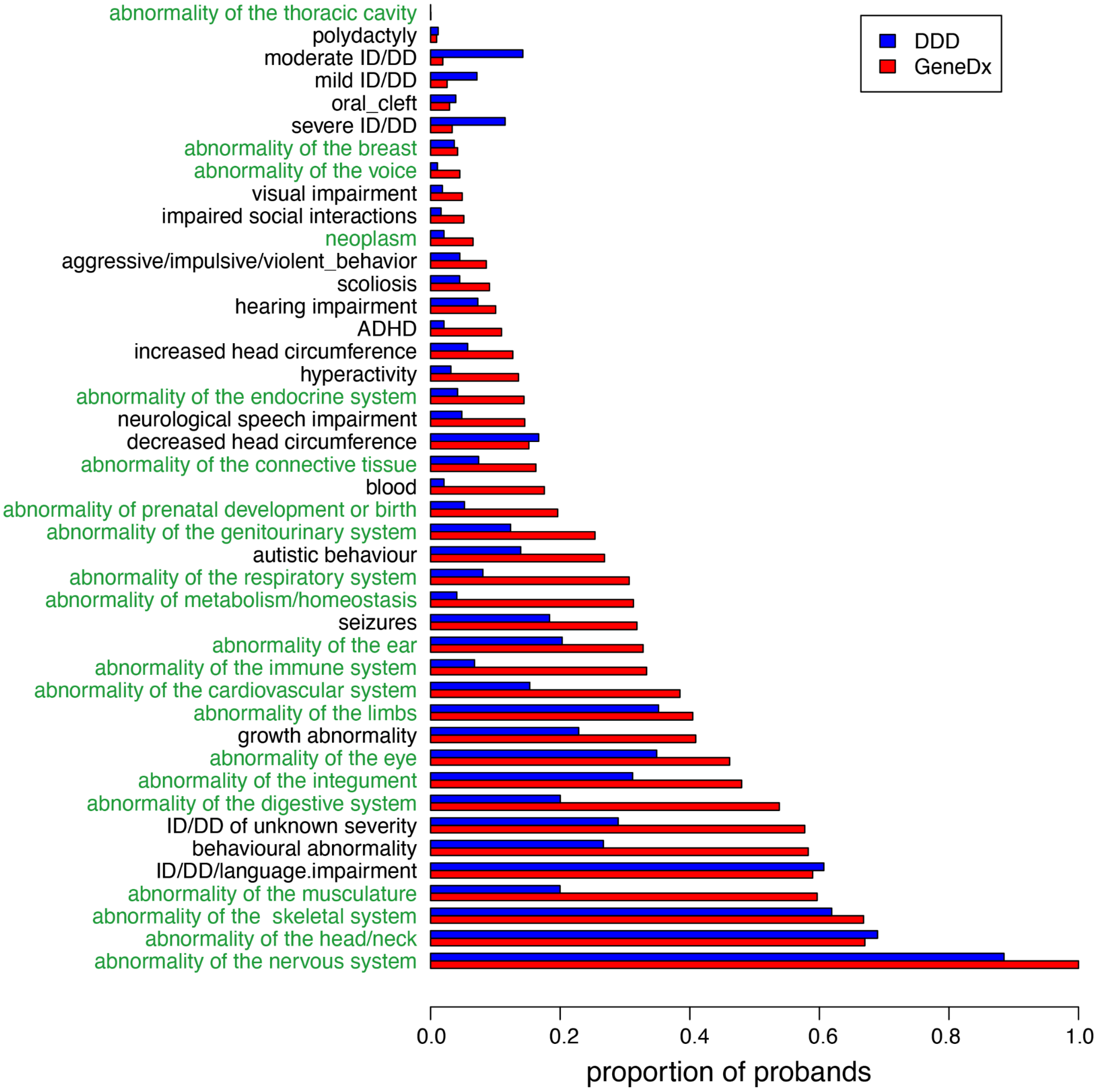

Supplementary Figure 2: Proportion of probands from each cohort with at least one HPO term within the indicated chapter (green text) or specific phenotype (black text), ordered by the prevalence in GeneDx. This is based on 13,450 DDD patients and 36,057 GeneDx patients. We used logistic regression to test whether there was a significant difference in phenotype prevalence between cohorts after controlling for sex and age. All of the indicated phenotypes showed a significant association with cohort (two-sided binomial $p<0.0001$, all passing Bonferroni correction for multiple comparisons) except the following: any ID/DD/language impairment, polydactyly, abnormality of the breast and abnormality of the thoracic cavity.

Supplementary Figure 3

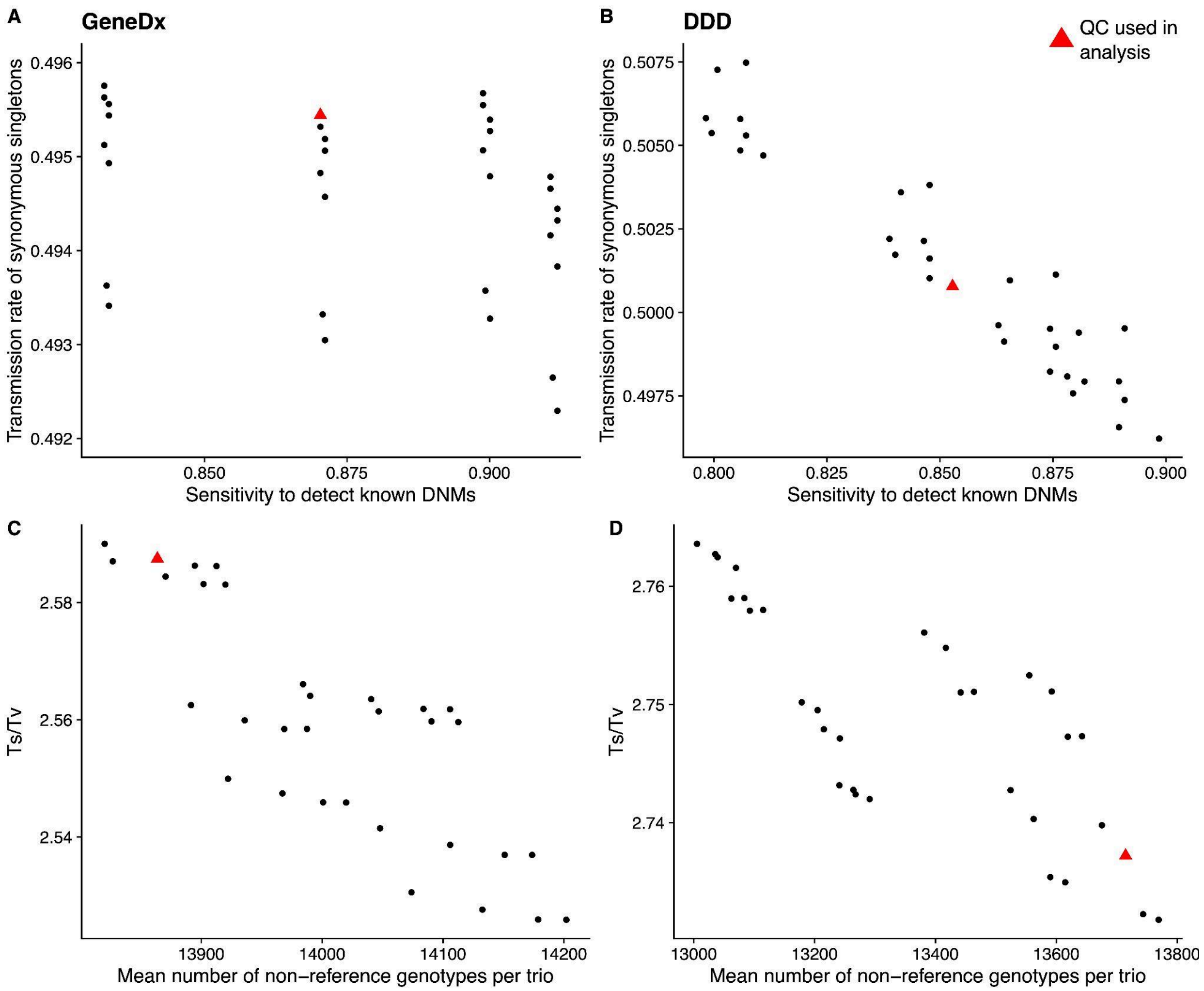

Supplementary Figure 3: Choosing optimal QC metrics for SNVs. A) and B) show the transmission rate of synonymous singletons vs sensitivity to detect validated *de novo* variants in GeneDx and DDD respectively. Panels C) and D) show the transition to transversion ratio versus the mean number of non-reference genotypes per trio in GeneDx and DDD respectively. The red triangle represents the value for the final QC threshold chosen.

Supplementary Figure 4

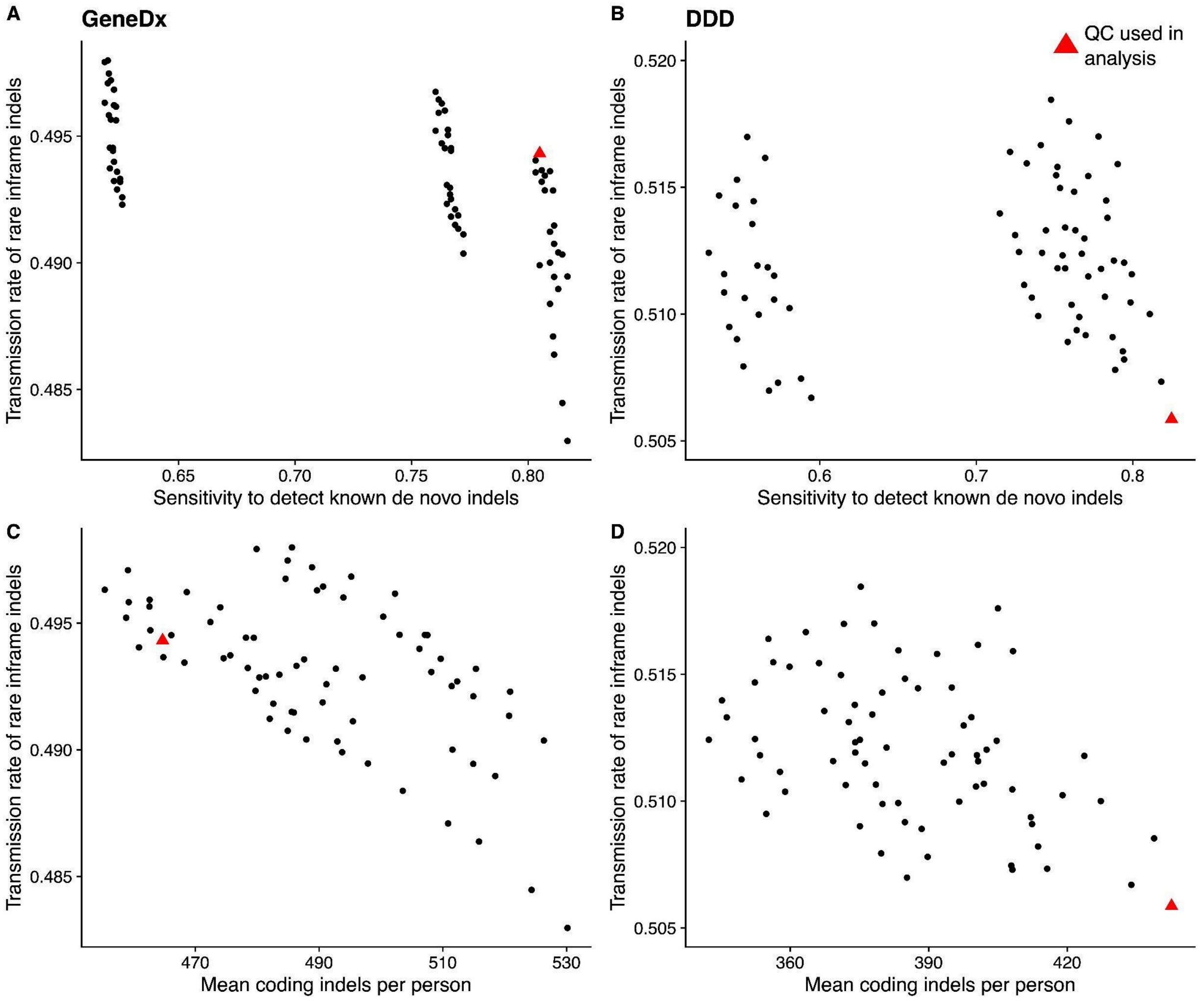

Supplementary Figure 4: Choosing optimal QC metrics for indels. A) and B) show the transmission rate of rare inframe variants in low pLI non-monoallelic DDG2P genes vs sensitivity to detect validated *de novo* indels in GeneDx and DDD respectively. Panels C) and D) show the same transmission rate as the previous panels versus the mean number of coding indels per person in GeneDx and DDD respectively. The red triangle represents the final QC threshold chosen.

Supplementary Figure 5

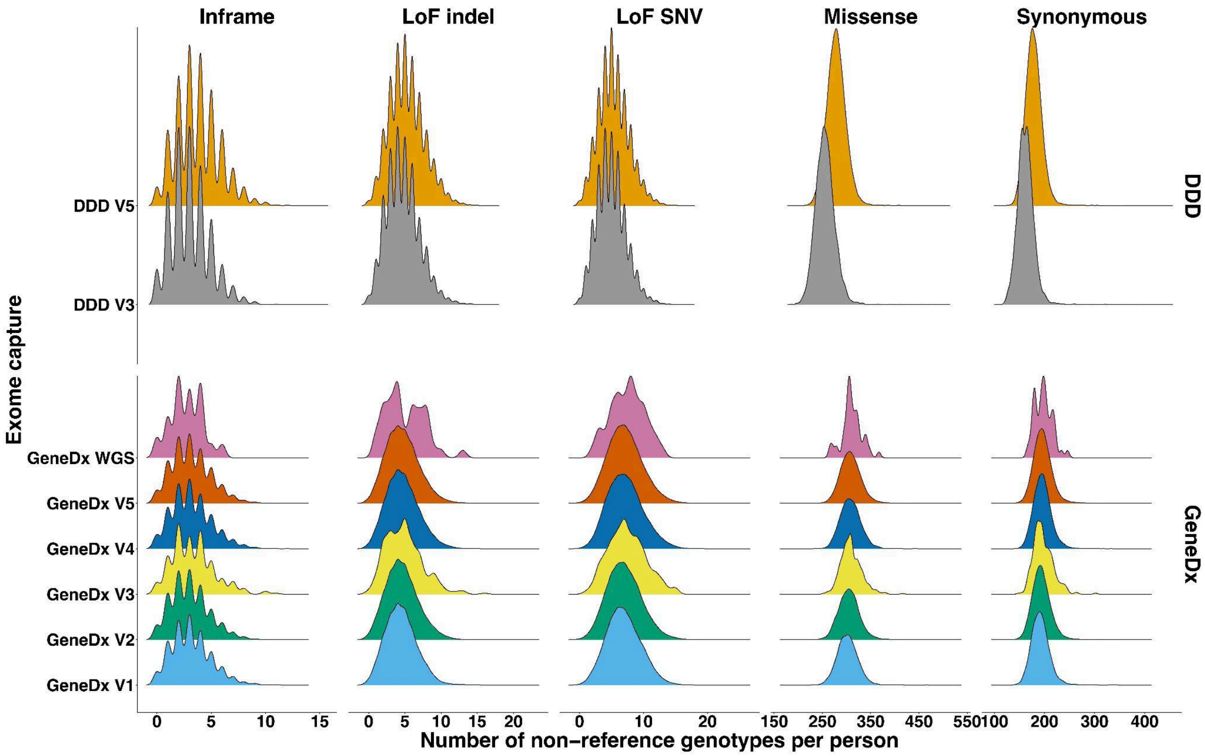

Supplementary Figure 5: Rare variant (MAF<0.01 across the cohort) count distributions for different exome capture platforms for EUR4 individuals.

Supplementary Figure 6

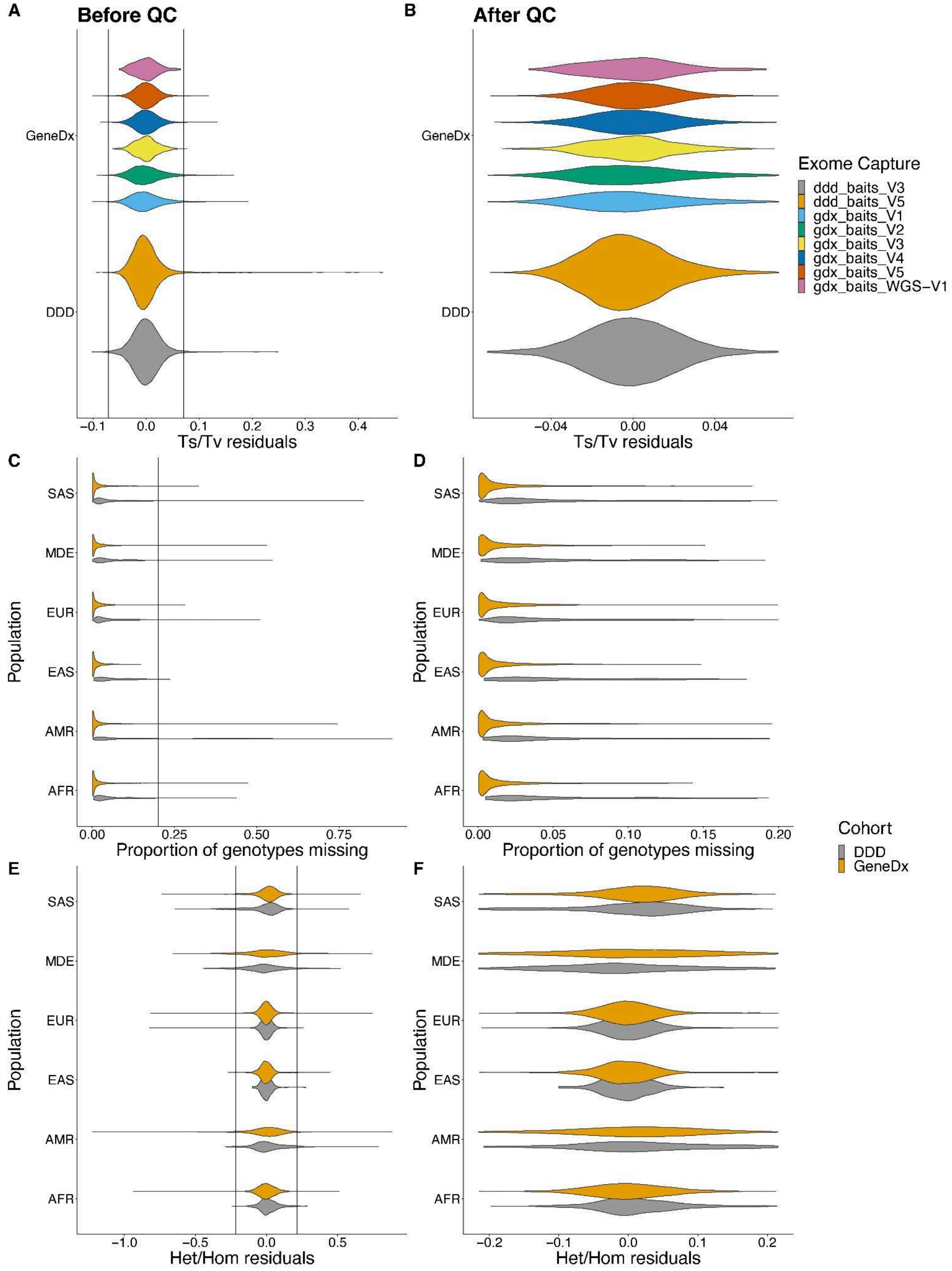

Supplementary Figure 6: Distribution of various metrics per sample before and after sample QC. A) The residuals of the transition-transversion ratio, after regressing out the effect of population, and exome capture, split by exome capture before removing sample QC outliers and B) after removing the sample QC outliers. C) The proportion of genotypes missing, split by dataset, before removing sample QC outliers, and D) after removing sample QC outliers. E) The residuals of the heterozygous to homozygous alternative ratio, after regressing out the effect of population, exome capture, and F_{ROH}, split by exome capture, before removing sample QC outliers, and F) after removing sample QC outliers. The vertical lines represent the thresholds for outlier removal (see Sample QC section of the Supplementary Methods for details).

Supplementary Figure 7

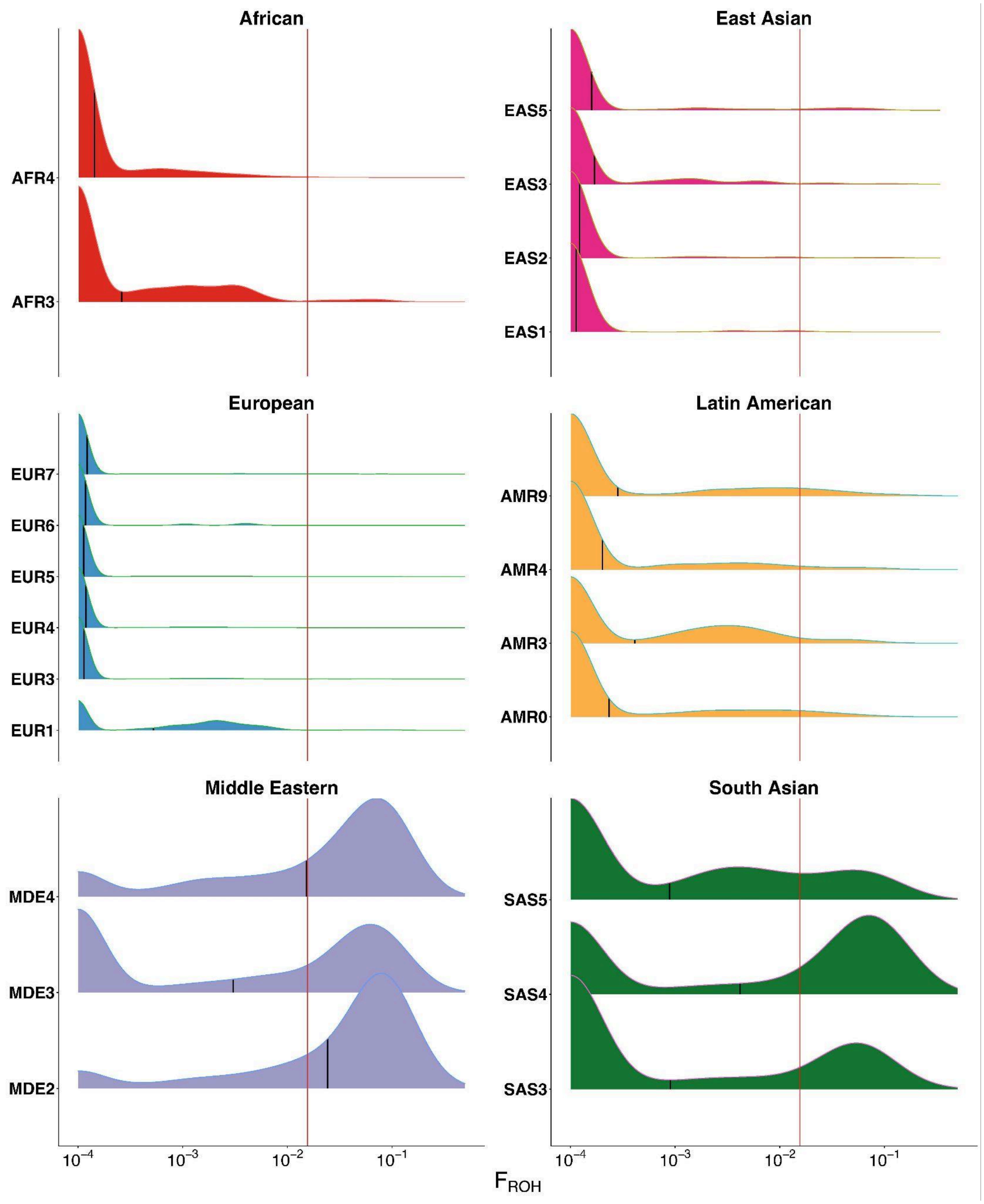

Supplementary Figure 7: Density plots of distribution of F_{ROH} amongst the probands per GIA sub-groups on a pseudo-log scale (F_{ROH}+0.0001). The vertical line represents F_{ROH}=0.0156 which is the expected value for the offspring of second cousins.

Supplementary Figure 8

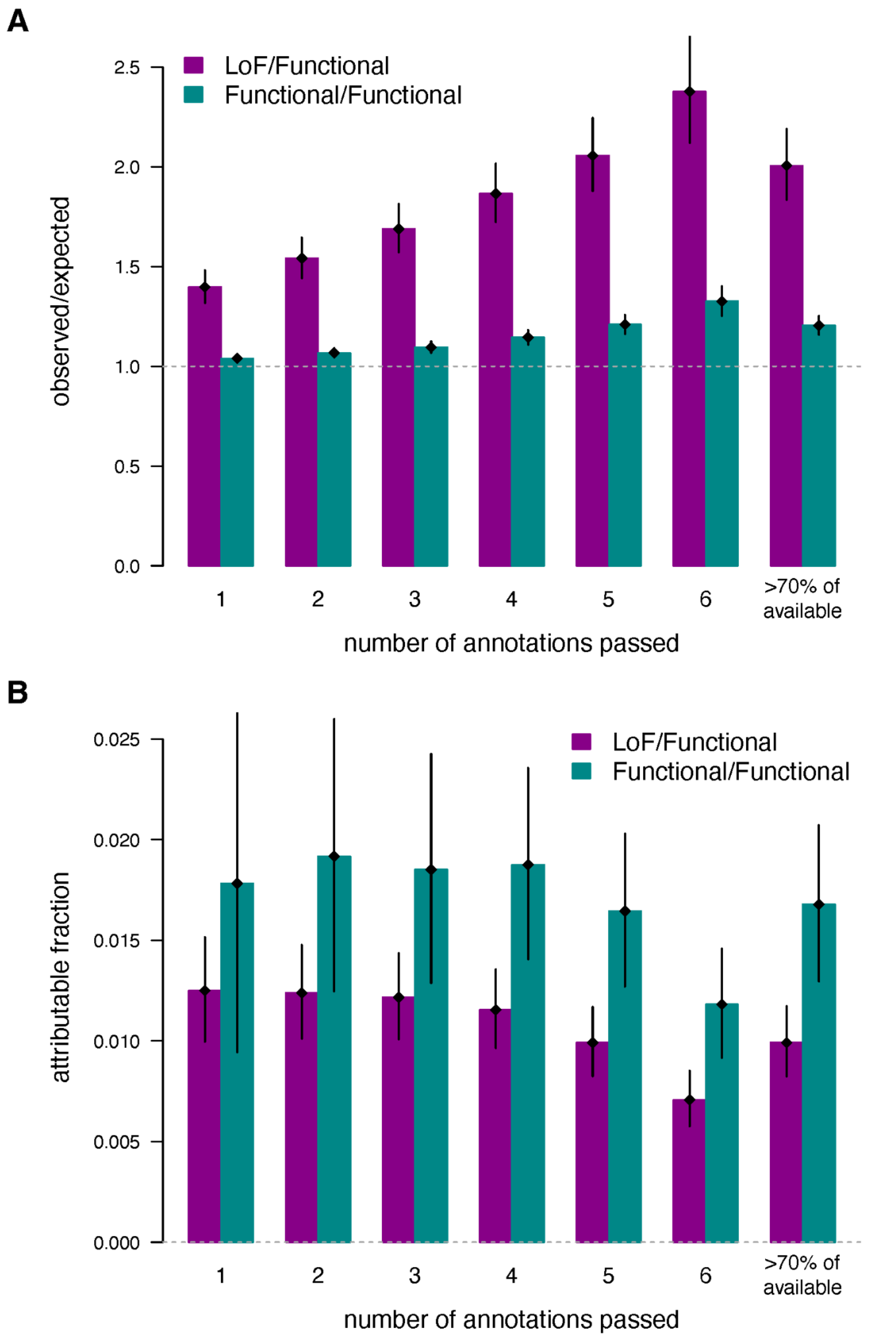

Supplementary Figure 8: Effect of different strategies for filtering missense variants on exome-wide burden and attributable fraction. A) Ratio of observed to expected genotypes and B) attributable fraction [(Observed-Expected)/N], for LoF/functional and functional/functional genotypes, using different numbers of missense pathogenicity filters (see section on “Filtering of missense and other functional variants” in the Methods). Results are from the same samples as Figure 1 (i.e., the seven large GIA sub-groups with the cross-continental admixture filter, for GeneDx and DDD combined, N=25,523). Error bars show 95% confidence intervals.

Supplementary Figure 9

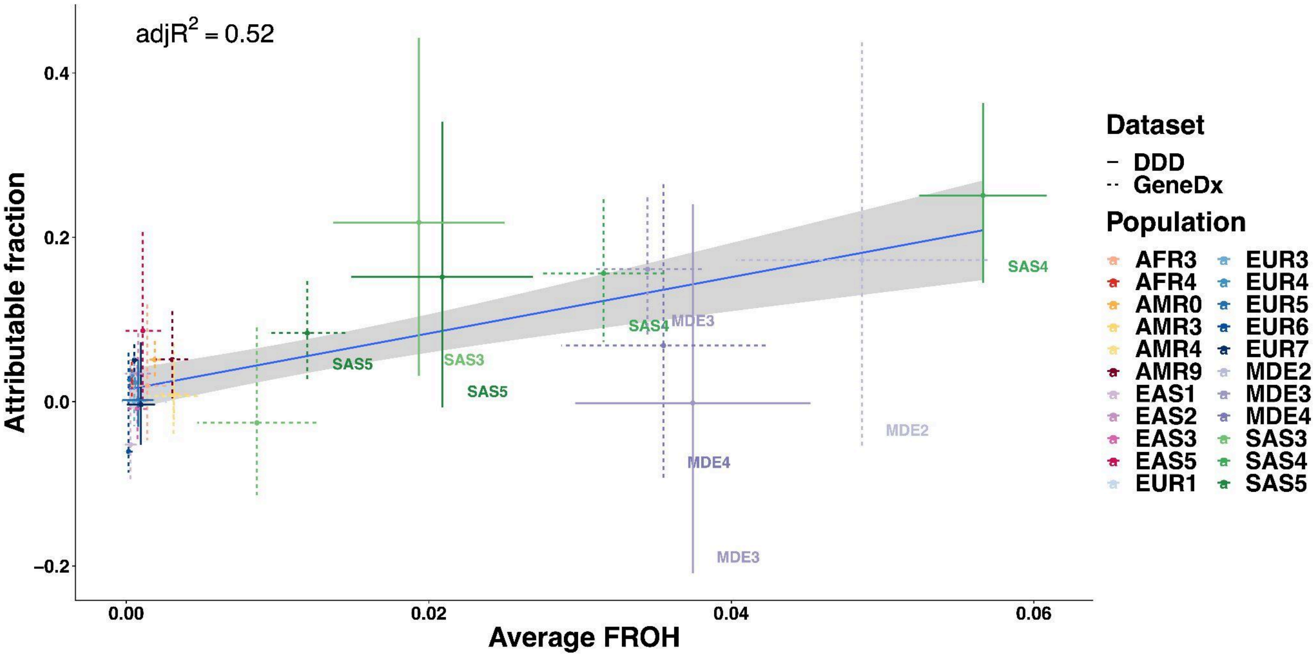

Supplementary Figure 9: The estimated attributable fraction versus the average F_{ROH} for all twenty-two GIA sub-groups included in Table 1, split by cohort. The line of best fit is shown, with a 95% confidence interval around it shown in grey shading, with an adjusted r^2 of 0.52. The centre of the error bars represents the point estimate for the attributable fraction (y-axis) and average F_{ROH} (x-axis) for that sub-group. Error bars on the points show 95% confidence intervals around these estimates.

Supplementary Figure 10

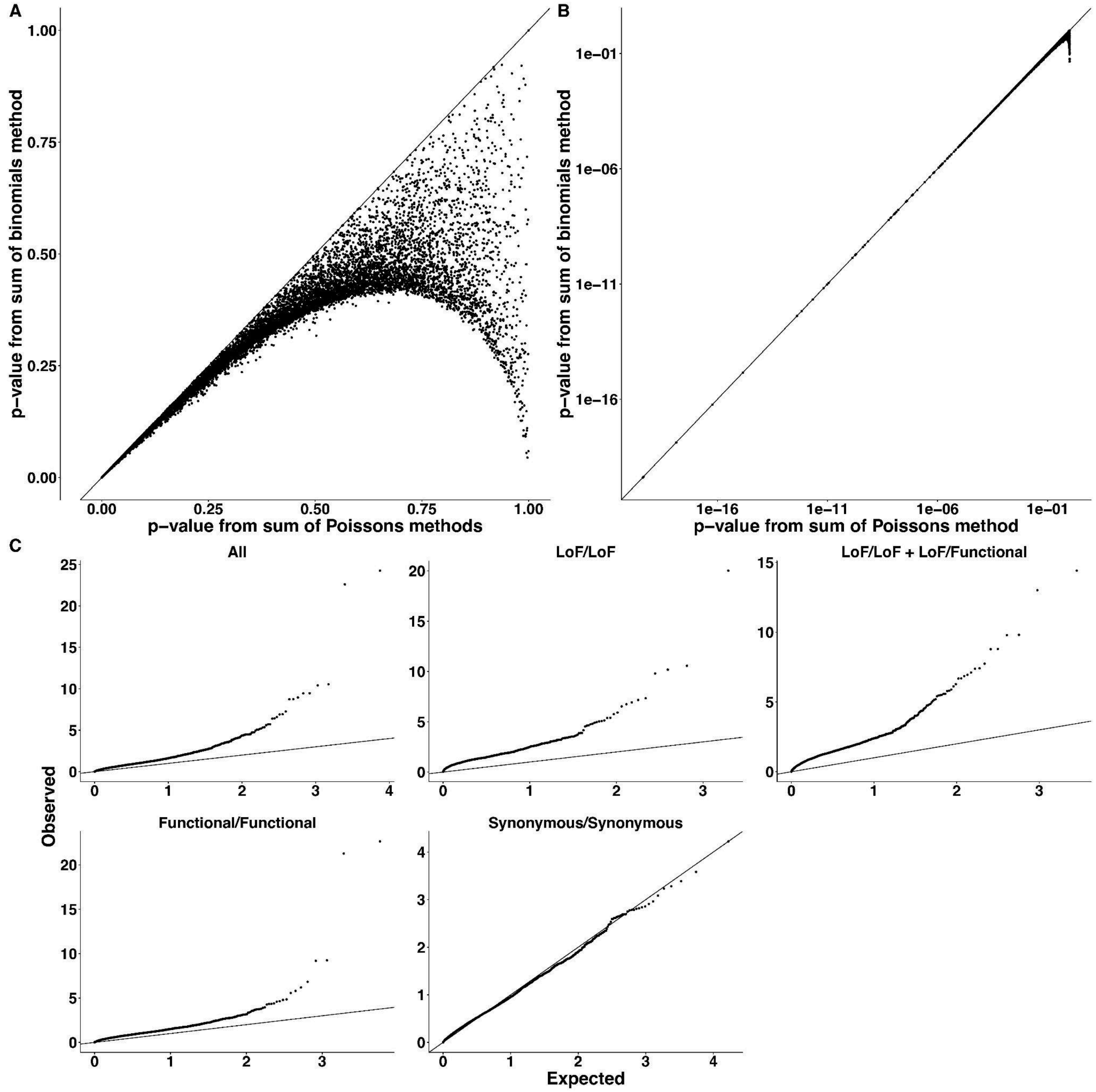

Supplementary Figure 10: Performance of the per-gene tests. A) and B) are a comparison of the p-values, across every consequence class (i.e., four tests per gene plotted here), from the sum-of-Poissons method (one-sided Poisson test) used in this paper with the sum-of-binomials method (equivalent to a one-sided binomial test) used in Akawi et al. [39] and Martin et al. [3]. These show that the methods agree well for low p-values (Pearson correlation of 0.98 for panel A). Panel C) shows QQ-plots of the sum-of-Poissons method applied to the genotypes in the indicated consequence classes, after removing genes with zero observed counts. These are based on 29,745 unrelated trios without cross-continental admixture from the twenty-two GIA sub-groups shown in **Table 1**.

Supplementary Figure 11

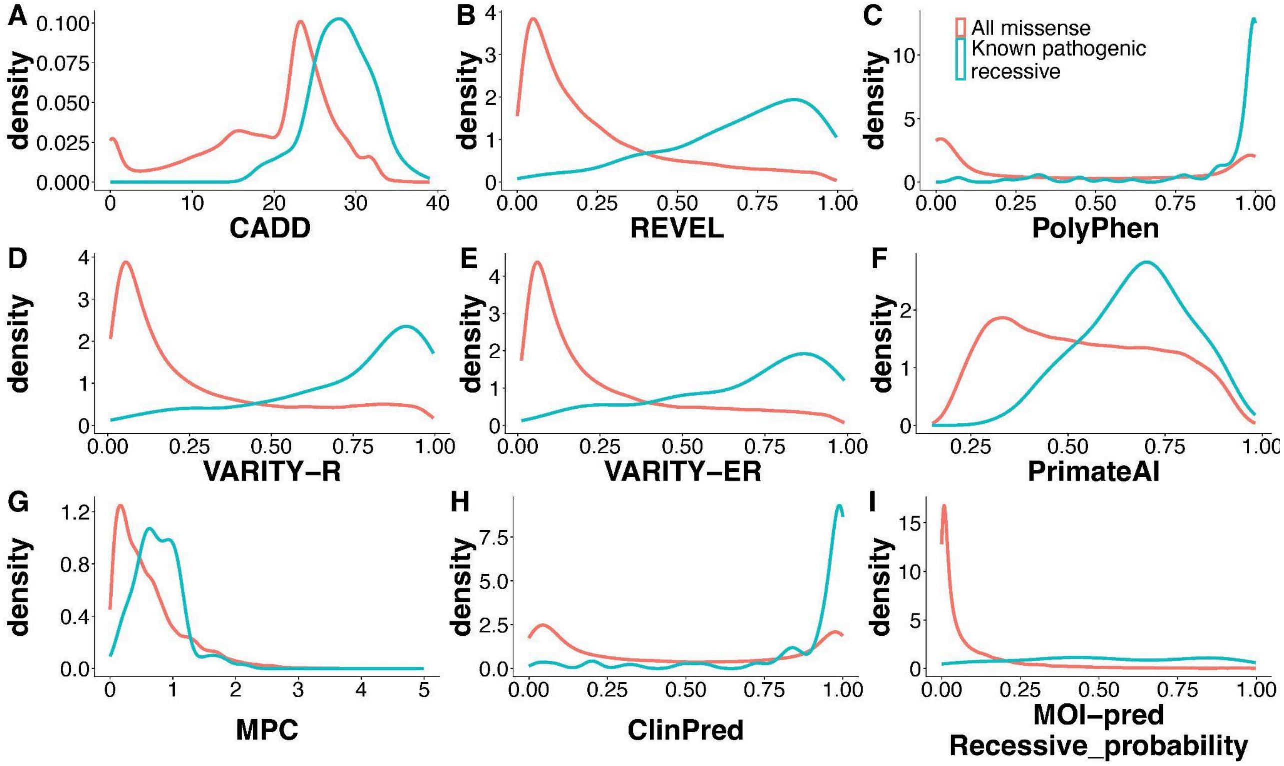

Supplementary Figure 11: Distributions of pathogenicity predictors for 122 known pathogenic recessive missense variants from DECIPHER versus all missense variants on chromosome 20.

Supplementary Figure 12

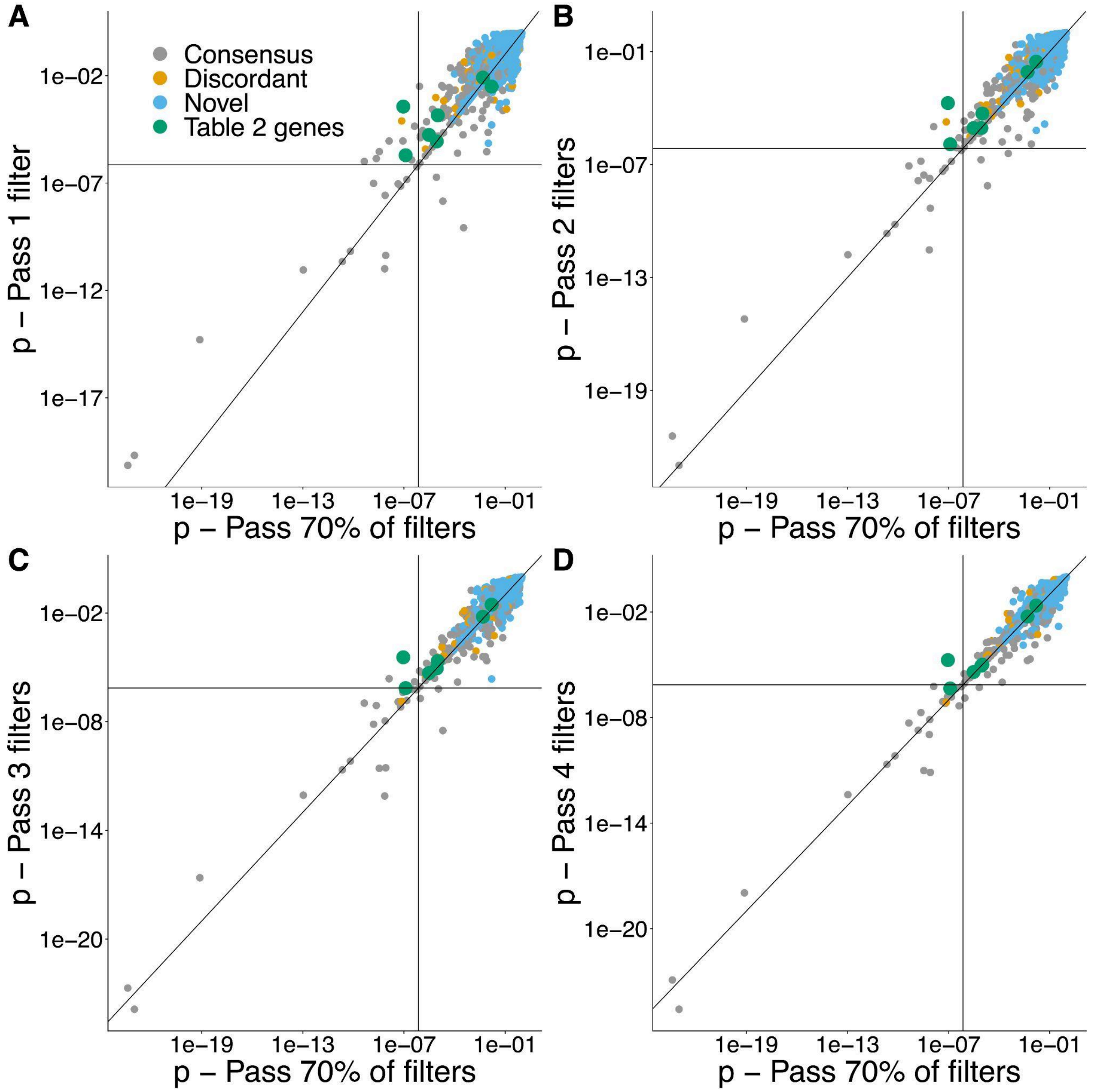

Supplementary Figure 12: Comparison of p-values from the per-gene tests (one-sided Poisson test) obtained using missense variants passing A) one, B) two, C) three or D) four deleteriousness filters versus passing 70% of the available missense deleteriousness filters (used in the main analysis). Only the p-value for the consequence combination that was most significant in the main analysis is shown. Genes highlighted in **Table 2** are coloured in green. No adjustment has been made to the p-value for multiple comparisons here.
