# Supplementary material for: Federated analysis of autosomal recessive coding variants in 29,745 developmental disorder patients from diverse populations
Source: Nat Genet. 2024 Sep 23;56(10):2046–53. doi: 10.1038/s41588-024-01910-8 (PMC11525179; doi:10.1038/s41588-024-01910-8)
Supplement: Supplementary file 2 — Reporting Summary [file 41588_2024_1910_MOESM2_ESM.pdf]

nature portfolio

Corresponding author(s): Hilary Martin

Last updated by author(s): Aug 4, 2023

Reporting Summary

Nature Portfolio wishes to improve the reproducibility of the work that we publish. This form provides structure for consistency and transparency in reporting. For further information on Nature Portfolio policies, see our Editorial Policies and the Editorial Policy Checklist.

Statistics

For all statistical analyses, confirm that the following items are present in the figure legend, table legend, main text, or Methods section.

n/a	Confirmed	
☐	☒	The exact sample size (*n*) for each experimental group/condition, given as a discrete number and unit of measurement
☒	☐	A statement on whether measurements were taken from distinct samples or whether the same sample was measured repeatedly
☐	☒	The statistical test(s) used AND whether they are one- or two-sided<br>*Only common tests should be described solely by name; describe more complex techniques in the Methods section.*
☐	☒	A description of all covariates tested
☐	☒	A description of any assumptions or corrections, such as tests of normality and adjustment for multiple comparisons
☐	☒	A full description of the statistical parameters including central tendency (e.g. means) or other basic estimates (e.g. regression coefficient) AND variation (e.g. standard deviation) or associated estimates of uncertainty (e.g. confidence intervals)
☐	☒	For null hypothesis testing, the test statistic (e.g. *F*, *t*, *r*) with confidence intervals, effect sizes, degrees of freedom and *P* value noted<br>*Give P values as exact values whenever suitable.*
☒	☐	For Bayesian analysis, information on the choice of priors and Markov chain Monte Carlo settings
☒	☐	For hierarchical and complex designs, identification of the appropriate level for tests and full reporting of outcomes
☐	☒	Estimates of effect sizes (e.g. Cohen's *d*, Pearson's *r*), indicating how they were calculated

Our web collection on statistics for biologists contains articles on many of the points above.

Software and code

Policy information about availability of computer code

Data collection

No software was used to collect data.

Data analysis

In DDD, the BWA aln algorithm (BWA version 0.5.10) and the BWA mem algorithm (BWA version 0.7.12) were used to align reads.Picard Markduplicates (versions 1.98 and 1.114)33 and Genome Analysis Toolkit IndelRealigner (GATK version 3.1.1 and version 3.5.0) were used for sample-level BAM improvement. GATK (version 3.5.0) HaplotypeCaller was used for variant calling.

In GeneDx, BWA (v0.5.8 to v0.7.8, depending on the time of sequencing) was used to align the reads. BAM files were then converted to CRAM format with Samtools version 1.3.139. Individual gVCF files were called with GATK v3.7-0 HaplotypeCaller. Multi-sample GVCF files were jointly genotyped using GATK v3.7-0 GenotypeGVCFs. GATK v3.7-0 VariantRecalibrator (VQSR) was applied for both SNPs and indels.

KING (version 2.2) was used to infer kinship. GCTA (version 1.93.0) was used to run principal component analysis, and HBDSCAN to determine fine- scale ancestry clusters. Variant annotation was carried out using VEP v94.5.

We made use of the following software and packages:

R	3.6.3(for analysis) & 4.1.3(for plotting)
Python	3.9.15
htslib	1.15.1
bcftools	1.16-63
r-dplyr	1.0.6
tabix	1.11
numpy	1.24.1

scipy	1.10.0
cyvcf2	0.30.16
pandas	1.5.2
pytabix	0.1
pyranges	0.0.120
umap-learn	0.5.3
hdbscan	0.8.29
r-ggplot2	3.4.2
r-dplyr	1.1.0
r-cowplot	1.1.1
r-data.table	1.14.6
r-ggridges	0.5.4
r-patchwork	1.1.2
plink	1.9

The code to perform the burden analysis and reproduce plots from this paper is available on GitHub (https://github.com/chundruv/ DDD_GeneDx_Recessives), as is the code to run the Phenopy method (https://github.com/GeneDx/phenopy).

We made use of data from gnomAD v2.1.1, 1000 Genomes Phase 3 and Human Genome Diversity Project (2019 release).

For manuscripts utilizing custom algorithms or software that are central to the research but not yet described in published literature, software must be made available to editors and reviewers. We strongly encourage code deposition in a community repository (e.g. GitHub). See the Nature Portfolio guidelines for submitting code & software for further information.

Data

Policy information about availability of data

All manuscripts must include a data availability statement. This statement should provide the following information, where applicable:

- Accession codes, unique identifiers, or web links for publicly available datasets
- A description of any restrictions on data availability
- For clinical datasets or third party data, please ensure that the statement adheres to our policy

Sequence and variant-level data and phenotype data from the DDD study data are available on the European Genome-phenome Archive (EGA; https://www.ebi.ac.uk/ega/) with study ID EGAS00001000775. The datasets most of interest for replicating findings in this study are EGAD00001004388, EGAD00001004389 and EGAD00001004390. GeneDx data cannot be made available through the EGA owing to the nature of consent for clinical testing. GeneDx-referred patients are consented for aggregate, deidentified research and subject to US HIPAA privacy protection. As such, we are not able to share patient-level BAM or VCF data, which are potentially identifiable without a HIPAA Business Associate Agreement. Access to the deidentified aggregate data used in this analysis is available upon request to GeneDx. GeneDx has contributed deidentified data to this study to improve clinical interpretation of genomic data, in accordance with patient consent and in conformance with the ACMG position statement on genomic data sharing. Clinically interpreted variants and associated phenotypes from the DDD study are available through DECIPHER (https://www.deciphergenomics.org/). Clinically interpreted variants from GeneDx are deposited in ClinVar (https://www.ncbi.nlm.nih.gov/clinvar) under organisation ID 26957 (https://www.ncbi.nlm.nih.gov/clinvar/submitters/26957/).

Research involving human participants, their data, or biological material

Policy information about studies with human participants or human data. See also policy information about sex, gender (identity/presentation), and sexual orientation and race, ethnicity and racism.

Reporting on sex and gender	Only one analysis in this paper (Supplementary Figure 11) is stratified by sex. It was stratified by genetically-determined sex. The purpose was to test for sex differences in the recessive burden of developmental disorders
Reporting on race, ethnicity, or other socially relevant groupings	In this work, we classified individuals into genetically-inferred ancestry (GIA) groups. The rationale for this was two-fold: firstly, we were interested in exploring differences in genetic architecture between these groups, and secondly, the analysis relies on accurate estimates of allele frequencies which differ between groups. The classifications were based on genetic similarity to individuals in the 1000 Genomes and Human Genome Diversity Panel (HGDP) reference datasets, inferred from principal component analysis. We defined six continental-level GIA groups (AFR: African; AMR: Latin American; EAS: East Asian; EUR: European; MDE: Middle Eastern; SAS: South Asian) and, within these, forty-seven fine-scale GIA sub-groups. The Methods are fully described in the manuscript, as is the justification for doing this.
Population characteristics	The individuals in this study are patients with neurodevelopmental disorders (or their parents) who were genetically undiagnosed prior to sequencing by the DDD study or GeneDx. For DDD, the average age was 7.3 (standard deviation 6.1 years). For GeneDx, it was 9.4 years (SD 10.2 years). 58.4% of DDD probands were male versus 55.7% in GeneDx.
Recruitment	Between April 2011 and April 2015, the DDD study patients a severe developmental disorder who remained undiagnosed after undergoing the typical clinical genetics investigations. The phenotypic inclusion criteria included neurodevelopmental disorders, congenital abnormalities, growth abnormalities, dysmorphic features, and unusual behavioural phenotypes. Recruitment took place across twenty-four regional genetics services within the United Kingdom and the Republic of Ireland health services. Since the study only included patients who were undiagnosed through the usual clinical means, it may be depleted of easy-to-diagnose recessive cases, as mentioned in the main text.<br>Patients were referred to GeneDx for clinical whole-exome sequencing for diagnosis of suspected Mendelian disorders, as described in https://www.nature.com/articles/gim2015148. Patients were selected for inclusion in this study based on having one or more HPO terms from a list of 716 that fell under “abnormality of the nervous system”.
Ethics oversight	The DDD study was approved by the UK Research Ethics Committee (10/H0305/83, granted by the Cambridge South Research Ethics Committee, and GEN/284/12, granted by the Republic of Ireland Research Ethics Committee). The GeneDx

study was conducted in accordance with all guidelines set forth by the Western Institutional Review Board, Puyallup, WA (WIRB 20162523).

Note that full information on the approval of the study protocol must also be provided in the manuscript.

Field-specific reporting

Please select the one below that is the best fit for your research. If you are not sure, read the appropriate sections before making your selection.

☒ Life sciences ☐ Behavioural & social sciences ☐ Ecological, evolutionary & environmental sciences

For a reference copy of the document with all sections, see nature.com/documents/nr-reporting-summary-flat.pdf

Life sciences study design

All studies must disclose on these points even when the disclosure is negative.

Sample size	We used the largest available sample size, combining data from two cohorts.
Data exclusions	We excluded individuals who did not have neurodevelopmental disorders, or who failed genetic quality control, as described in the Methods section.
Replication	We did not replicate as each disorder is very rare, as such we did not have another dataset to use. We did however find extra cases for the significant genes
Randomization	No randomization was needed. We also did not control for covariates (e.g. genetic principal components), but rather restricted the calculation of allele frequencies to genetically homogeneous subgroups.
Blinding	Not applicable since no specific grouping.

Reporting for specific materials, systems and methods

We require information from authors about some types of materials, experimental systems and methods used in many studies. Here, indicate whether each material, system or method listed is relevant to your study. If you are not sure if a list item applies to your research, read the appropriate section before selecting a response.

Materials & experimental systems

n/a	Involved in the study	
☒	☐	Antibodies
☒	☐	Eukaryotic cell lines
☒	☐	Palaeontology and archaeology
☒	☐	Animals and other organisms
☒	☐	Clinical data
☒	☐	Dual use research of concern
☒	☐	Plants

Methods

n/a	Involved in the study	
☒	☐	ChIP-seq
☒	☐	Flow cytometry
☒	☐	MRI-based neuroimaging
